# The use of recombinant morphogenic protein-2(rhBMP-2) in children undergoing revision surgery for persistent non-union

**DOI:** 10.1007/s11751-016-0251-9

**Published:** 2016-03-16

**Authors:** Madhavan C. Papanna, K. A. Saldanha, Binu Kurian, James A. Fernandes, Stan Jones

**Affiliations:** Department of Trauma and Orthopaedics, Sheffield Children Hospital, Sheffield, S10 2TH UK

**Keywords:** Bone, Congenital abnormalities non-union, rhBMP-2

## Abstract

The purpose of the study was to evaluate the safety and efficacy with the use of BMP-2 for treating persistent non-unions in children with underlying complex conditions. Between October 2006 and November 2010 in our unit, 15 patients were treated with rhBMP-2 to enhance bone union. There were nine females and six males with a mean age of 9.5 years (range 4–15) at time of surgery. Seventy-five per cent of the patients required revision of internal fixation with insertion of rhBMP-2 to the non-union site, and the reminder had freshening of the non-union site with rhBMP-2 application. Patients had undergone a mean of 2 (1–5) operations prior to implantation of rhBMP-2. All the patients in the study group were available for review with mean follow-up of 44 months (range 21–70). The mean time to union was 16 weeks (range 10–28 weeks). No adverse events related to BMP-2 application were noted in our study group. Healing occurred clinically and radiographically in 16 of the 17 sites. Our study demonstrates that BMP-2 enhances healing of the persistent non-unions without any adverse events

## Introduction

Autologous bone grafting (ABG) has osteogenic, osteoinductive and osteoconductive properties and is the gold-standard biological treatment for non-union [1, 2]. However, limited availability and donor site morbidity limit its use [3, 4]. In 1965, Marshal R Urist discovered a substance within the extracellular matrix of bone that induced new bone formation when implanted into extraskeletal sites in a host. This substance triggers a proliferation of undifferentiated mesenchymal cells and the formation of osteoprogenitor cells to form bone. It was called bone morphogenic protein (BMP). By 1988, molecular clones had been characterised and the amino acid sequence from a highly purified bovine bone preparation was derived. This led to the isolation of human complimentary DNAs, recognised subsequently as a member of the superfamily of transforming growth factor β. At least 20 human variants of BMPs that possess varying degrees of osteoinductive activity have been identified since [5].

Two (BMP-2 and BMP-7) have been the subject of intense research for treatment of non-union and are available currently as recombinant protein molecules of human genes [5]. The Food and Drug Administration (FDA) and the European agency for the evaluation of medical products have approved the use of BMP-2 as bone graft substitute in adults with open tibial fractures and those undergoing anterior lumbar inter-body spinal fusion as an adjunct to standard care by internal fixation [6–10].

In addition to the approved use, there have been reports of use in an off-label fashion in children undergoing surgery for spinal and orthopaedic conditions [11–13, 18, 19]. However, there are limited published data on the use and outcomes of BMP-2 in revision non-union surgery in the paediatric population.

In children, fractures and corrective osteotomies heal well mostly. However, union may be difficult to achieve in patients with skeletal dysplasias, congenital deficiencies of the limbs and some complex fractures. This is our experience with the use of BMP-2 in children undergoing revision surgery for persistent non-union.

## Materials and methods

We undertook a retrospective review of all the patients who received rhBMP as a part of their treatment at the Sheffield Children’s Hospital between October 2006 and November 2010. This review was approved by the research and development department of our institution. In all patients, the decision to use rhBMP-2 was made at a multidisciplinary team meeting. We had approval from the hospital pharmacy department and also obtained informed consent from the parents of our patients for the use of rhBMP-2.

Clinical data for each patient were gathered from the medical records and included demographics, anatomical site, diagnosis, initial treatment, number and type of previous operations, operative details at the time of rhBMP-2 use, time to union and the length of follow-up (see Table 1).

**Table 1:** Demographics and clinical profile of the patients

Case number	Age (years)/gender	Diagnosis	Anatomical site	Type of non-union	Previous surgical treatment	Number of previous surgeries	Revision surgical procedure	Number of application sites	Time to healing (weeks)	Outcomes	Follow-up (months)
1	M,4	PFFD	Femur	Oligotrophic	Hybrid frame application	1	Revision IF	1	22	Healed	26
2	F,4	PFFD	Femur	Oligotrophic	Proximal femoral osteotomy and acetabuloplasty	1	Revision IF and debridement of non-union site	1	24	Healed	30
3	F,11	OI type 3	Femur	Oligotrophic	Growing rod insertion	2	Revision IM nail	1	16	Healed	42
4	F,12	(Perthes disease/down’s syndrome)	Femur	Oligotrophic	Pelvic supportive osteotomy + femoral lengthening with hybrid frame application	3	Freshening non-union site	2	28	Healed	25
5	F,10	Non-union achondroplasia	Femur	Oligotrophic	Intramedullary nail insertion	2	Revision fixation	1	20	Healed	22
6	M,13	OI type 4	Tibia	Oligotrophic	Growing rod insertion	3	Revision growing rod	1	10	Healed	21
7	M,12	CPT	Tibia	Oligotrophic	Growing rods +bone grafting application	5	Revision to growing rod	1	14	Healed	33
8	M,15	Fracture nonunion (coats plus disease)	Tibia	Atrophic	Taylor spatial frame application	3	Freshening non-union site	1	N/A	No (awaiting revision surgery)	44
9	F,4	CPT	Tibia	Oligotrophic	Nancy nails insertion	4	Revised to growing rod	1	12	Healed	70
10	F,12	OI type 3	Bilateral tibia	Oligotrophic	Growing rods insertion	2	Revision growing rod	2	18	Healed	34
11	F,13	OI type 3	Tibia	Oligotrophic	Growing rod insertion	1	Revision growing rod	1	26	Healed	24
12	F,14	Non-union (closed fracture)	Tibia	Oligotrophic	Taylor spatial frame application	1	Freshening non-union site	1	12	Healed	41
13	M,15	OI type 4	Ulna	Oligotrophic	Corrective osteotomy and intramedullary nail	2	Revision IM nail	1	12	Healed	36
14	M,12	Malunited monteggia fracture with radial head dislocation	Ulna	Oligotrophic	Intramedullary nail insertion	1	Corrective ulna osteotomy + TSF Application	1	10	Healed	38
15	F,9	Arthrogryposis with fixed equinus	Ankle	Oligotrophic	Arthrodesis of the ankle with screws	1	Freshening of non-union site	1	8	Healed	46

*OI* osteogenesis imperfecta, *PFFD* proximal femoral focal deficiency, *CPT* congenital pseudoarthrosis of the tibia, *TSF* Taylor spatial frame, *IM* intramedullary nail, *IF* internal fixation

Nineteen patients (21 surgical procedures) received rhBMP-2 as a part of their treatment during the study period. Four patients were excluded as they were either older than 18 years, had autologous bone graft in addition to rhBMP-2 or had a spinal fusion procedure. The final sample was comprised of 15 patients (17 surgical procedures). Case 4 required two episodes of rhBMP-2 application to a femoral non-union site and case 10 had bilateral application of rhBMP-2 to tibial non-union sites at different stages. The mean age of these patients at the time of rhBMP-2 use was 9.5 years (range 4–15 years). Nine were female and six male (Table 1).

All the patients had a persistent non-union or pseudoarthrosis despite previous surgery to achieve union. With the exception of one case (case 8) that was an atrophic non-union, the remainder had radiographic features of oligotrophic non-union (Table 1).

The patients had undergone a mean of 2 (range 1–5) previous surgical procedures prior to the use of rhBMP-2. The surgical procedures included resection of pseudoarthrosis and autologous bone grafting in 10 patients (62 %), intramedullary fixation with rods, fixation with a plate and screws or application of external fixator.

The predominant primary diagnosis was osteogenesis imperfecta (5 patients). The other diagnoses were proximal femoral focal deficiency (2 patients), neurofibromatosis with pseudoarthrosis of the tibia (2 patients), non-union after comminuted fractures (2 patients), achondroplasia, arthrogryposis, Coats’ plus disease and a femoral fracture in a patient with both Down’s syndrome and Perthes disease.

The senior authors (JAF and SJ) evaluated patients for clinical evidence of healing by pain and tenderness at the non-union site and the ability to weight bear on the affected limb with the orthosis. The radiographs were evaluated independently for any complications and signs of healing. Friedlander’s criterion (the presence of bone bridging at the site of non-union in at least one view) was used [20]. The non-union was considered healed if it fulfilled radiological and clinical criteria.

### Operative technique

All the surgical procedures were performed under general anaesthetic. Prophylactic antibiotic was administered at the time of induction and two further doses given at 8 and 16 h postsurgery. Using a tourniquet, the non-union site was exposed through a longitudinal skin incision. Fibrous tissue and avascular bone were excised until healthy bone ends were exposed. In some cases of tibial non-union, it was necessary to undertake a fibular osteotomy, done through a separate lateral skin incision.

The next stage of the surgery involved a revision of the fixation device if required. For intramedullary nails, the medullary canal of the proximal and distal segments was drilled with increasingly larger drill bits to accommodate the larger nails. In those patients with external fixators in situ, these were adjusted accordingly and some compression applied.

BMP-2 was reconstituted with sterile water to a concentration of 1.5 mg/ml and a bovine collagen sponge used as delivery matrix. After at least 15 min of soak time and just before closure of the surgical wound, the BMP-2-impregnated sponge was cut into rectangular pieces and implanted directly over the bone ends. Demineralised bone matrix (DBX) was placed over the BMP-2 in patients with large defects. The amount of BMP-2 used was determined by the size of the bone cavity or defect. Autologous bone graft was not used in any of the cases.

All the patients were allowed to commence partial weight bearing once the surgical wound had healed. Clinical and radiological follow-up was undertaken at regular intervals until union was achieved.

## Results

None of the patients was observed to have a septic non-union. At the time of revision surgery with rhBMP-2, 75 % of the patients required revision of the previous fixation device. Twelve patients required revision fixation at the time of BMP-2 insertion that included Sheffield telescopic rods for the tibia and femur in five and two patients, respectively, whereas Fassier–Duval telescopic rods were used in the tibia of two patients. In two patients with femoral non-unions, plates were used, and in one patient, an Ilizarov ring fixator was used to stabilise the femur.

**Fig. 1:**
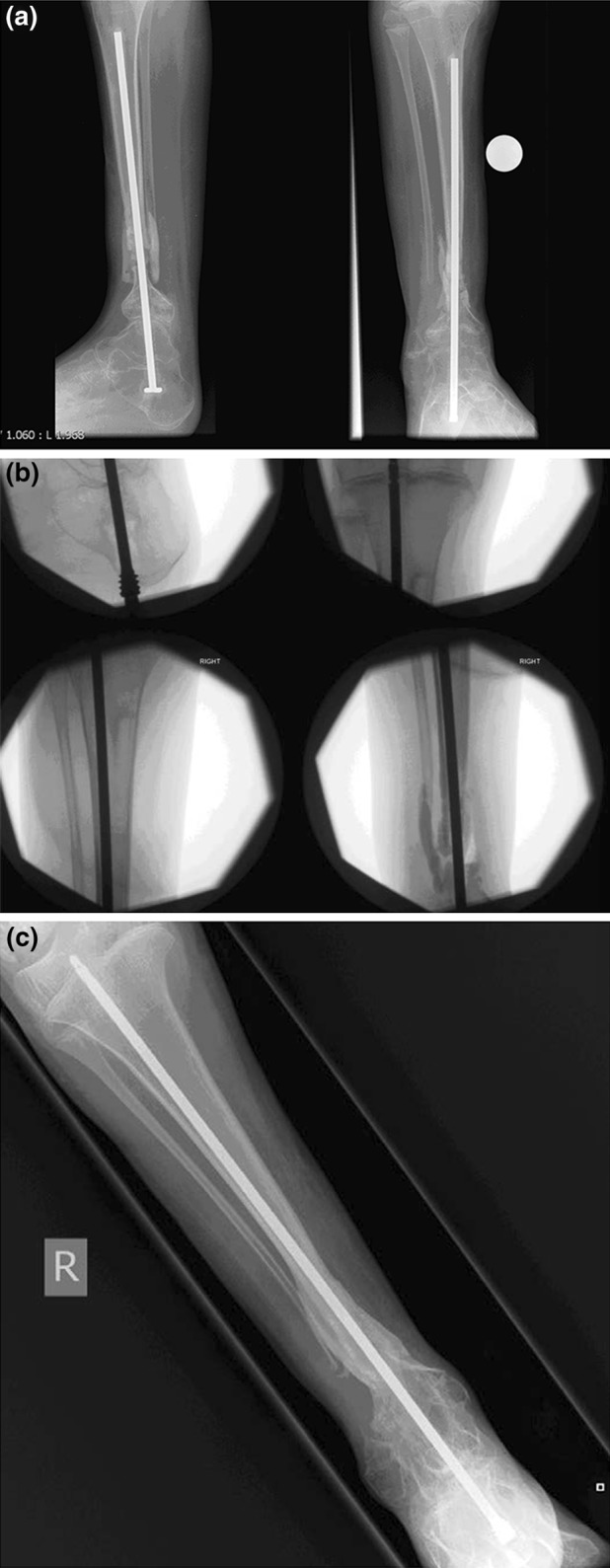
This 12-year-old patient with congenital pseudoarthrosis of the tibia had multiple surgical procedures to enhance the healing of non-union. **a** Preoperative radiograph showing the non-union of the pseudoarthrosis with growing rod in situ. **b** Intraoperative images illustrating the revision intramedullary nailing and BMP-2 insertion. **c** Anteroposterior view radiograph at 14 weeks after initial surgery showing healing at the pseudoarthrosis site

All the patients in the study group were available for review at a mean follow-up of 44 months (range 21–70). The mean time to union was 16 weeks (range 10–28 weeks) (Fig. 1a, c). Clinical and radiological healing was observed in 16 of the 17 sites at the last follow-up. One patient (case 8) with Coats’ plus disease was treated with BMP-2 and an Ilizarov fixator for tibial non-union 10 months after the index surgery and failed to heal. Further autologous bone grafting was performed, and at 6 months postoperatively the bone has failed to unite and the patient is awaiting further surgery.

No local or systemic complications attributable to BMP-2 were noted in any of our patients. In particular, none of our patients had a wound breakdown, local soft tissue calcification or heterotrophic ossification.

## Discussions

Bone morphogenic proteins possess good osteoinductive properties that enhance healing and are used in the treatment of adult patients with recalcitrant non-unions and spinal fusion procedures successfully to facilitate union/fusion [7, 8, 20]. The manufacturers of commercially available recombinant human BMP-2 have stated that it is contraindicated for use in the paediatric population because they have not been able to provide data that establish the safety and efficiency of BMP-2 in children below 18 years of age. There have been reports of use of BMPs in the paediatric population [11–19] with most as case reports [13, 14] and small case series [15, 17–19]; the prevalent clinical condition for its use was congenital pseudoarthrosis of the tibia [12, 15–19].

In comparison, use of rhBMP-7(OP-1) for treating non-union and congenital pseudoarthrosis of the tibia in the paediatric population [14–17] had mixed success. Lee et al. reported on five patients with congenital pseudoarthrosis of the tibia treated using bone graft, rhBMP-7 and fixation. Union was achieved in only one of the five cases, and it was felt that variables in the surgical technique contributed to the poor outcome [15]. Other authors have reported reasonable outcomes [14, 16–18]. The results of these studies suggest that rhBMP-7 should be combined with autologous bone graft and optimum fixation of the pseudoarthrosis is required.

The current literature describes rhBMP-2 used mostly for the treatment of congenital pseudoarthrosis of tibia in the paediatric population [18, 19]. Spiro et al. [18] reported four children with congenital pseudarthrosis of the tibia treated with intramedullary stabilisation, Ilizarov external fixators and rhBMP-2. Only one out of four had previous failed surgery. Radiological union was achieved at a mean of 3.5 months postoperatively with a mean follow-up of 31 months. They concluded that the initial rate of union may be improved and the time to union reduced with this strategy. Richards et al. [19] reported on seven children with CPT treated using rhBMP-2, autologous bone graft and intramedullary rodding. Two patients had failed previous surgery. Radiological union was achieved in five patients at a mean of 6.4 months. The average follow-up was 72 months, and no adverse effect of BMP was observed. They also noted an improvement in the time to initial union. Their average of 6.4 months compared favourably with 16 months reported by Dobbs et al. [22] who treated a similar group of patients using autologous bone graft and intramedullary rodding but without BMP.

In this series, we observed a mean time to union of 16 weeks. This compares favourably with the reports of Spiro et al. (14 weeks) and Richards et al. (26 weeks). This may be because most of our cases were not congenital pseudoarthroses of the tibia. The time to union of the two cases of CPT in this study was 12 and 14 weeks, respectively. The non-unions in this series were due to multiple factors, viz. biology and stability. RhBMP-2 is not effective in the presence of instability at the non-union site. The one patient in this study who failed union despite using rhBMP-2 and an Ilizarov fixator was a case of Coats’ plus disease with a tibial non-union (case 8). Further autologous bone grafting failed, and further surgery is being planned. We believe the failure to achieve healing is related to the underlying diagnosis and not surgical technique. It is established that congenital defects decrease fusion rates [1].

This report contains the second largest number of patients (15 patients) but with a longer follow-up than that published by Oetgen et al. Fifty-three of 81 patients in their series were skeletally immature, and BMP-2 was used mostly as part of spinal surgery. The report was focussed on the complications associated with the use of BMP-2 [12], citing an overall complication of 17.5 % in 81 patients. The complications included excessive wound discharge and swelling, wound dehiscence, deep infection, enlargement of optic glioma, compartment syndrome, progressive myelopathy and dural fibrosis. They believed that only one of the complications may have been directly related to the use of BMP-2; this was dural fibrosis associated with motor weakness after exposure of the spinal cord to rhBMP-2 [12].

Ritting et al. [13] reported a case of massive inflammatory reaction following the use of rhBMP-2 to treat an ulnar non-union in a child. Circulating antibodies against type 1 collagen and anti-BMP-2 antibodies have been detected in a smaller number of patients treated with BMP, but these studies have concluded that there is insufficient evidence to establish a relationship between these antibodies and the absence of ossification [6, 20, 21].

Although there is a theoretical risk of adverse events in association with the use of BMP in skeletally immature patients, this is not confirmed in the literature. In the follow-up period of this study, we did not observe any local or systemic adverse events related directly to the use of BMP-2. The patients and the families in this study were warned of the risk of developing adverse effects and complications such as deep infection, a severe inflammatory reaction, neuralgia, resorption of bone, compartment syndrome, heterotrophic ossification and local nerve compression.

There are limitations to this study. This is a retrospective review of a small sample described by the common feature of having had failed attempts to treat a non-union. The sample was heterogeneous and without a control group for comparison. Alteration to the biomechanics (adjustment of fixation method) across the non-union would have influenced the results as would have use of the rhBMP.

In conclusion, this review describes successful use of rhBMP-2 as a part of a treatment strategy for persistent non-unions in children who have failed to achieve bone healing despite standard methods of treatment.
